# Case Report: Usefulness, effectiveness, and safety of an advanced hybrid closed-loop system in a child with early identification of type 1 diabetes

**DOI:** 10.3389/fendo.2025.1598264

**Published:** 2025-06-03

**Authors:** Chiara Mameli, Maddalena Macedoni, Francesca Redaelli, Agnese Petitti, Adelina Hajro, Alessandra Bosetti, Gianvincenzo Zuccotti

**Affiliations:** ^1^ Department of Pediatrics, Vittore Buzzi Children’s Hospital, Department of Biomedical and Clinical Science, Università di Milano, Milan, Italy; ^2^ Department of Biomedical and Clinical Sciences, “L. Sacco”, Biological Chemistry and Nutritional Biochemistry Lab, Università di Milano, , Milan, Italy

**Keywords:** type 1 diabetes, screening, CGM, CSII, children

## Abstract

The usefulness, effectiveness, and safety of advanced hybrid closed-loop systems (AHCLs) in early-stage 3 type 1 diabetes (T1D) are unknown. We report 9 months of continuous glucose monitoring (CGM) outcomes in a patient with early-stage 3 T1D treated with the Tandem t:slim Control-IQ^®^ system. A 13-year-old girl, affected by celiac disease and Hashimoto’s thyroiditis, was diagnosed with T1D without symptoms (fasting c-peptide: 1.77 ng/ml; HbA1c: 6.4%) following an outpatient T1D screening program. She wore a CGM at diagnosis to closely monitor her glucose profile. After 6 months, when the time in range (TIR) fell below 80%, the Tandem t:slim Control-IQ^®^ was initiated. Standardized CGM metrics, as well as instances of severe hypoglycemia and diabetic ketoacidosis (DKA), were recorded. CGM data guided the initiation of early insulin therapy. Tandem t:slim Control-IQ^®^ system proved effective from the onset of T1D, with a low insulin requirement (0.1 U/kg/day, < 10 units/day) and maintained good metabolic control (TIR > 80%) without severe hypoglycemia or DKA. Based on our experience, we suggest a two-step approach for monitoring and treating patients with early stage 3 type 1 diabetes: first, positioning CGM, and second, when TIR falls below 80%, considering the addition of an AHCLs, even if the patient has a low insulin requirement.

## Introduction

The diagnosis of type 1 diabetes (T1D) in presymptomatic stages remains rare (1). Without screening, most diagnoses are made at stage 3, when symptoms appear due to hyperglycemia, and up to 40% of cases are diagnosed during diabetic ketoacidosis (DKA) (1, 2). Currently, no data are available about the fluctuation of blood glucose levels over 24 h, as measured by continuous glucose monitoring (CGM) in the earliest stages of the disease, particularly when diabetes-related symptoms are absent. These data could be instrumental in guiding timely detection and the initiation of prompt therapy of T1D, leading to substantial improvements in diabetes management and future care.

The role of advanced hybrid closed-loop systems (AHCLs) in diabetes is expanding. AHCLs have been shown to be superior to insulin pump therapy, sensor-augmented pumps, and multiple daily insulin in most studies involving the pediatric population (3). However, data on the usefulness and effectiveness of AHCLs in the early stages of diabetes, when insulin requirements are low and residual insulin secretion is present, are still lacking.

In 2023, the Italian Parliament approved a law (Italian Republic Law 130/2023) introducing nationwide screening for T1D and coeliac disease in the general population aged 1–17 years, as part of a public health program aimed at reducing the impact of these chronic diseases (4). Despite this important initial step, several key questions remain, particularly concerning how to manage early diagnoses.

We report the case of a pubertal Caucasian 13-year-old girl, diagnosed with early stage 3 T1D during a screening program, who presented without diabetes-related symptoms. She used CGM during the first 6 months of the disease, followed by an AHCL (Tandem t:slim Control-IQ ^®^ and Dexcom G6^®^). We reported 9 months of CGM data (time in range (TIR), time above range (TAR), and time below range (TBL)), as well as safety outcomes (severe hypoglycemia, DKA).

## Results

A pubertal 13-year-old girl (weight: 54 kg; BMI: + 0.47 standard deviation score (SDS)), affected by celiac disease since age 9 and Hashimoto’s thyroiditis since age of 11 and treated with levotyroxine (1.2 mg/kg/day) orally, presented a fasting glucose level of 114 mg/dl detected during her annual routine screening, in the absence of diabetes-related symptoms. No family history of autoimmune disorders, including T1D, was reported.

**Figure 1:**
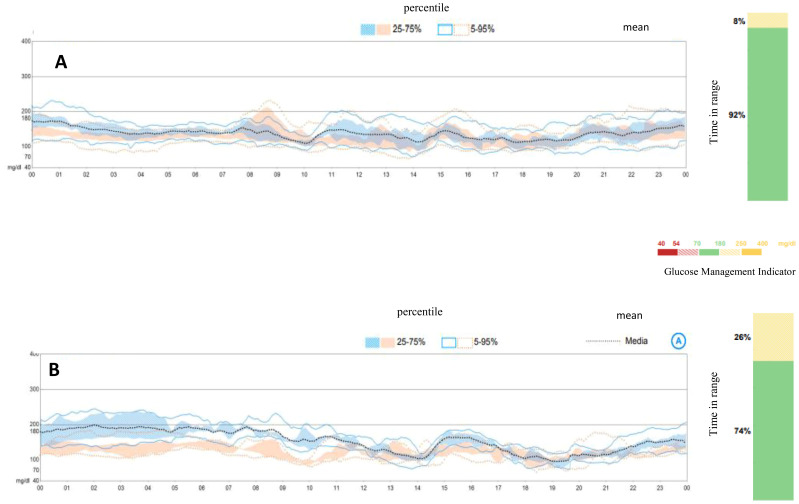
Ambulatory glucose profile and standardized CGM metrics before initiating AIDs. **(A)** Ambulatory glucose profile at diagnosis of early stage 3 type 1 diabetes. **(B)** Ambulatory glucose profile at 6 months from diagnosis, at the initiation of Tandem t:slim Control-IQ.

An oral glucose tolerance test showed a fasting blood glucose level of 113 mg/dl and a 2-h plasma glucose level of 278 mg/dl. HbA1c was 6.4% (46 mmol/mol). Two out of three diabetes-related autoantibodies were positive: GAD-ab: 19.23 U/ml (> 10 positive), ZNT8-ab: 1,760 U/ml (> 15 positive), ab anti-insulin: 2.23 U/ml (> 10 positive). Fasting c-peptide was 1.77 ng/ml. The girl was provided a Guardian™ Sensor 3 Medtronic^®^ and was educated by a diabetes specialist nurse to monitor her glucose profile at home. Two weeks later, CGM data showed a time in range of 92%, TAR 180–250 of 8%, TAR > 250 mg/dl of 0%. HbA1c was 6.3% (45 mmol/mol) (**Figure 1A**). Time in tight range (70–140 mg/dl) was 72%, and TAR > 140 mg/dl was 28%. Considering the 24-h profile and CGM metric, we decided to proceed with follow-up visits without initiating insulin therapy, while continuing patient-centered education on the disease and use of technology. During follow-up, CGM showed a decrease in TIR and an increase in TAR (**Figure 1B**). Six months later, postprandial and overnight hyperglycemia (glycemia > 200 mg/dl) were frequently detected by CGM and confirmed by capillary blood glucose measurements, with a reduction in TIR to < 80% (74%) and a TAR > 26% (26% in the 180–250-mg/dl range) (**Figure 1B**). Upon observing the worsening of the 24-h glucose profile, she was promptly admitted to the Pediatric Emergency Room—without DKA—and subsequently to the Pediatric Clinic to receive personalized education in disease management, carbohydrate counting, and use of technology from a diabetes specialist nurse and dietitian. HbA1c was 6.4% (46 mmol/mol). Tandem t:slim Control-IQ^®^ and Dexcom G6^®^ were initiated at 0.1 U/kg/day (insulin requirement: 4.5 insulin units/day; insulin correction factor: 1:500; carbohydrate ratio: 1:130; sleep activity enabled; weight: 54.1 kg; BMI: + 0.47 SDS). During the 3-month follow-up, TAR gradually increased to 94%. In parallel, TAR decreased to 6% (with > 250 mg/dl accounting for 2%), and no hypoglycemic events occurred (TBL: 0%) throughout the period. After 3 months of AHCL use, HbA1c was 6.7% (50 mmol/mol). The insulin requirement decreased to 0.07 units/kg/day, corresponding to 3.6 U/day. The CGM active time was 99.5%, with 99.8% in automode.

**Figure 2:**
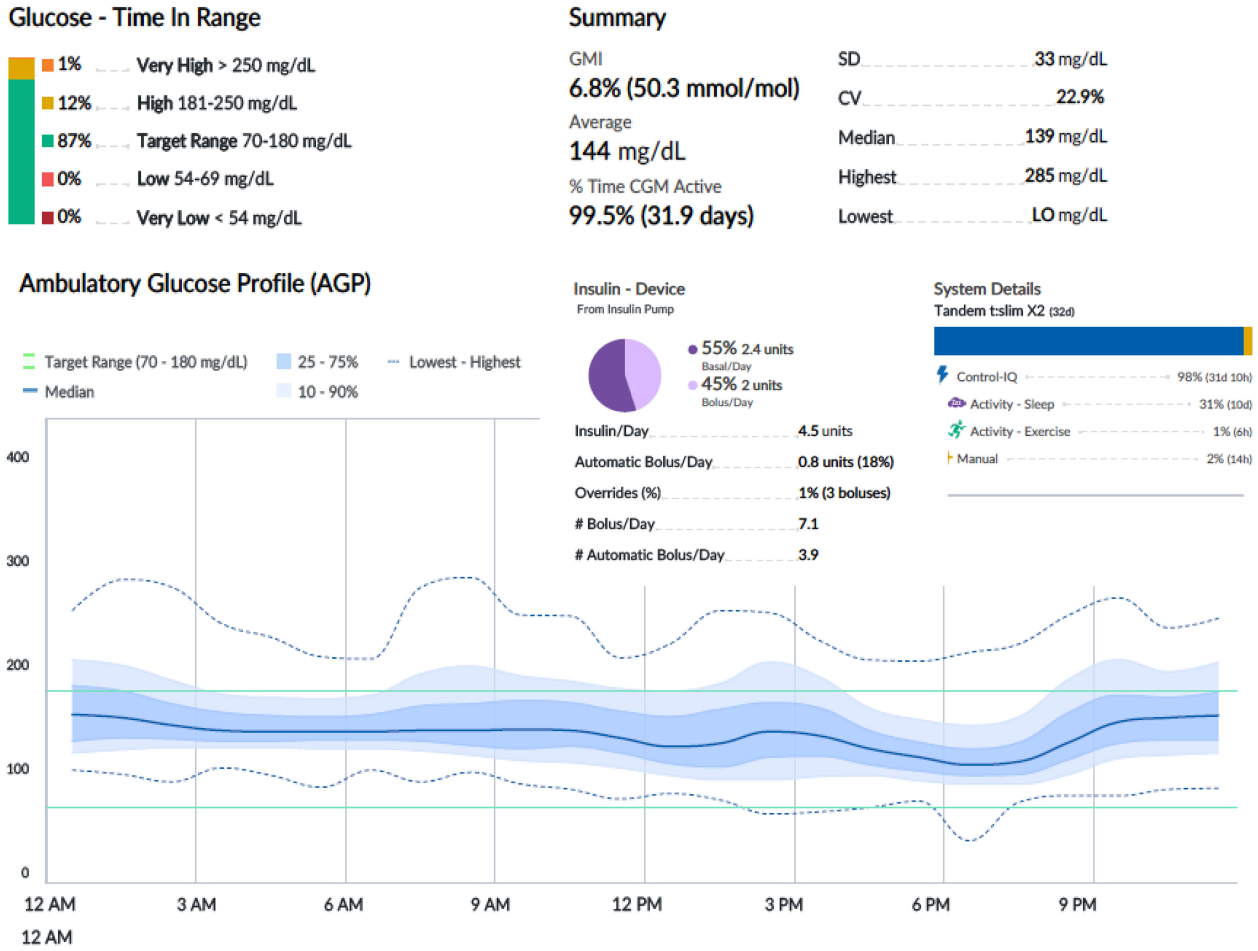
Month 1. Tandem Control IQ ambulatory glucose profile and metrics.

**Figure 3:**
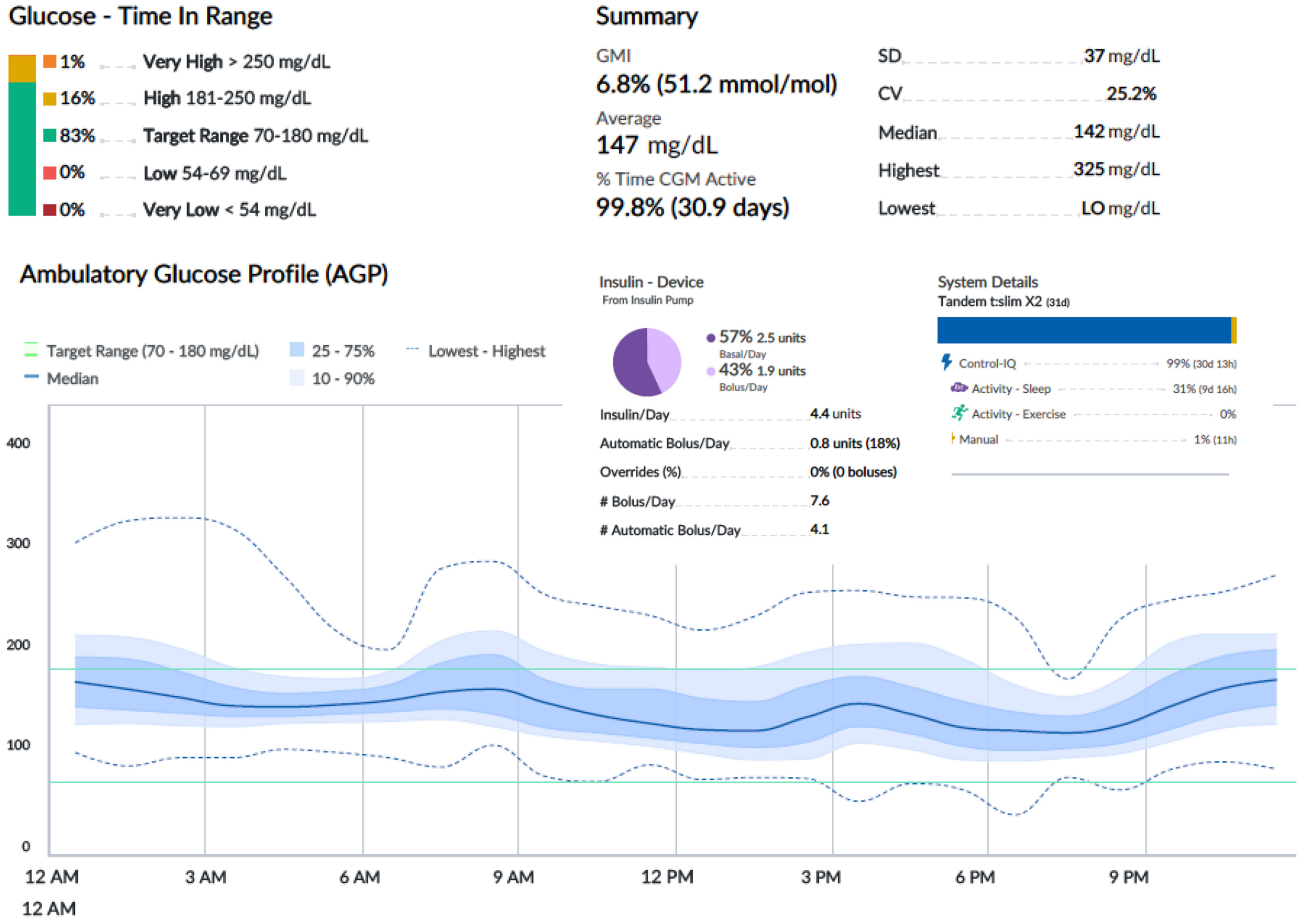
Month 2. Tandem Control IQ ambulatory glucose profile and metrics.

**Figure 4:**
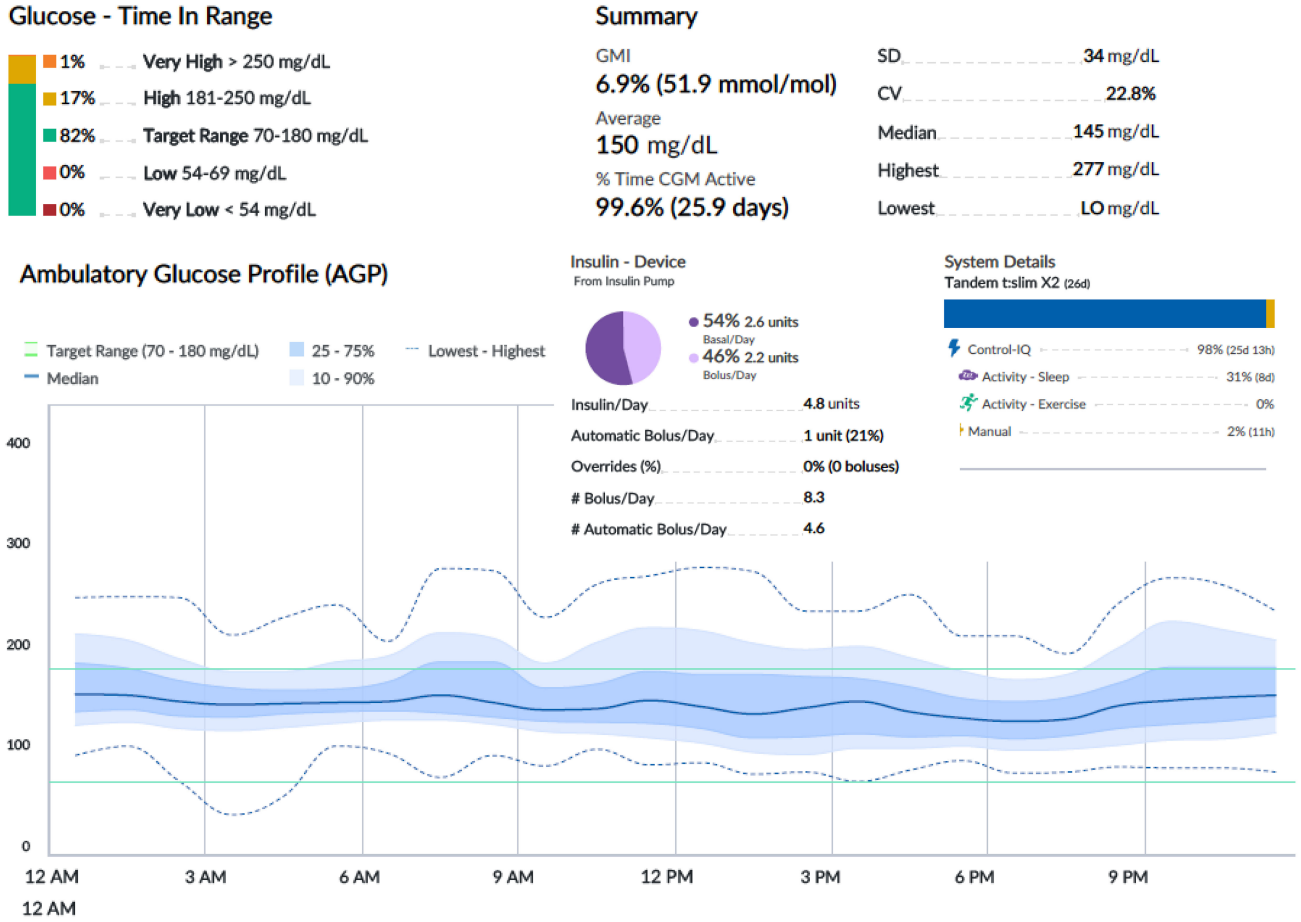
Month 3. Tandem Control IQ ambulatory glucose profile and metrics.

**Figures 2**-**4** show 30-day ambulatory glucose profiles during the first months of follow-up, indicating optimal metabolic control (TIR > 80%) throughout the period. No episodes of severe hypoglycemia or diabetic ketoacidosis were observed.

## Discussion

In this report, we describe the use of CGM and the Tandem t:slim Control-IQ^®^ system in early stage 3 T1D. Recent advancements have highlighted the utility of CGM systems for monitoring the early stages of T1D (5). Based on our experience, CGM was effective in monitoring the 24-h glucose profile and provided valuable information for initiating insulin therapy. A time-in-range of 80% was chosen as the cutoff for starting insulin therapy based on our clinical experience. The use of the Tandem t:slim Control-IQ ^®^ system from the onset of the disease aligns with the most recent consensus guidelines, which recommend that youth be offered the most advanced insulin delivery technology that is available, affordable, and appropriate (6). In our patient, the Tandem t:slim Control-IQ^®^ system effectively maintained good metabolic control, despite a low daily insulin requirement, generally between 4 and 5 units/day. Notably, based on our experience, the Tandem t:slim Control-IQ^®^ system functions effectively even when the total daily insulin dose is less than 10 units, without any safety concerns. No episodes of severe hypoglycemia, DKA, or other symptoms of diabetes were observed during the 3-month follow-up. A limitation of this approach is the direct comparison of CGM metrics before (Guardian 3) and during AHCL (Dexcom G6), although both sensors offer high accuracy and are approved for use in patients with T1D. Finally, the approach of first positioning CGM and then introducing AHCL in the early stages of the disease facilitates gradual patient education on the use of technology, which is essential in reducing future dropout.

The advent of screening for T1D presents the opportunity to diagnose the condition at earlier stages (stages 1 and 2) before the clinical disease onset (stage 3) (7). Recently, a consensus guideline was published, defining the staging criteria of T1D, the associated risk of progression, and the purpose of monitoring for any child or adolescent who has tested positive for islet autoantibodies (7). Monitoring for individuals in prestage 1 and stages 1–3 of T1D is determined by islet autoantibody status, glycemic status, diabetes-related symptoms, and insulin requirement. Individuals diagnosed before stage 3 T1D require ongoing monitoring for disease progression, including regular assessments of glucose levels (7). The earlier the diagnosis is made in the disease process, the sooner interventions can be initiated to prevent hyperglycemia. It is well known that maintaining blood glucose levels as close to normal as possible is essential for delaying or preventing complications (8). Of outstanding importance, individuals diagnosed with stage 2 T1D should be offered the opportunity to participate in clinical trials and receive access to disease-modifying agents or approved therapies aimed at delaying or halting disease progression (9).

Introducing technology earlier than usual in T1D management may increase the initial cost of diabetes care (10). However, considering the positive impact of AHCLs and CGMs on T1D health outcomes, these short-term costs may be offset by long-term savings. Comprehensive cost-effectiveness analyses are needed to support optimal T1D care in an era of increasing reliance on therapeutic technology and expanding T1D screening efforts.

## Conclusion

Based on our experience, the described two-step approach to monitoring and treating presymptomatic type 1 diabetes proved useful and effective, with no safety issues observed. First, CGM is initiated, and the patient is educated on glucose monitoring. Second, if TIR drops below < 80%, the use of an advanced AHCL should be considered, even in patients with low insulin requirements. It is important to note, however, that the findings from this case report cannot be generalized to all individuals with early-stage 3 T1D, as they are based on the clinical experience of our research group. We recommend that all treatment options be thoroughly discussed and that patient-centered care be prioritized.

## Data Availability

The raw data supporting the conclusions of this article will be made available by the authors, without undue reservation.
